# Exploring and validating themes of the eHealth Therapeutic Alliance Inventory in digital health interventions: A qualitative analysis

**DOI:** 10.1016/j.invent.2025.100895

**Published:** 2025-12-02

**Authors:** Ohad Ashur, Chen Rayan Saar, Or Brandes, Amit Baumel

**Affiliations:** a Department of Community Mental Health, University of Haifa, Israel

**Keywords:** Digital health, Therapeutic alliance, Mental health, eHealth, Qualitative study

## Abstract

**Objective:**

Studies indicate that users can establish a therapeutic alliance (TA) with digital health interventions. However, research examining how TA unfolds in digital settings and its unique facets compared to conventional TA remains limited. This qualitative study aimed to explore and validate preliminary quantitative findings suggesting three distinct themes unique to eHealth TA.

**Methods:**

Qualitative data were collected from users of a digital parent training program aiming to explore participants' general experiences with the program. Semi-structured double-blind interviews were conducted (*n* = 16), and responses to open-ended questions from a larger sample of users were collected (*n* = 64). Data were thematically analyzed using the six-step framework outlined by Braun and Clarke.

**Results:**

In the deductive thematic analysis all three eHealth TA themes were identified – Sense of Relatedness (SR), Application-Induced Accountability (AIA), and Perceived Emotional Investment (PEI), along with the three sub-themes of conventional TA (Goal, Task and Bond). Two eHealth TA themes (SR, AIA) were fully represented in participant's responses, while one (PEI) was only marginally validated. Regarding conventional TA, Goal and Task were fully represented, while Bond was only marginally validated. Validation of eHealth TA themes was further supported by the analysis of the open-ended questions.

**Conclusions:**

Findings reinforce prior research, indicating that TA in digital health settings unfolds in distinct ways, and underscoring the need for further exploration of eHealth TA's unique characteristics. Additionally, the study emphasizes potential advantages of employing double-blind methodology in qualitative studies.

## Highlights

• Qualitative validation confirms distinct eHealth Therapeutic Alliance facets, supporting earlier quantitative findings.
• This study only partly validated the therapeutic bond construct, indicating a need for further investigation in digital settings.
• Participants emphasized accountability and relatedness constructs over emotional investment, suggesting need for further exploration.

## 1 Introduction

### 1.1 Background

A growing body of research has emphasized the importance of therapeutic alliance (TA) in digital health interventions (Baumel et al., 2017; Berger, 2017; Cavanagh and Millings, 2013; Wehmann et al., 2020). Studies suggest that TA can be effectively established even within fully automated, unguided interventions (Lopez et al., 2019; D'Alfonso et al., 2020; Hollis et al., 2018). Notably, TA formed with conversational agents has been found to resemble the alliances formed between individuals and psychotherapists (Darcy et al., 2021; Gupta et al., 2022). However, while TA in traditional in-person settings has been extensively studied through various theoretical models and empirical investigations (Flückiger et al., 2018; Horvath et al., 2011; Elvins and Green, 2008), research into how TA manifests in digital health environments, along with the identification of its unique components, remains underexplored (Stern et al., 2024; Tong et al., 2022; Tremain et al., 2020). A significant gap in the field is that many existing eHealth TA measures simply adapt traditional, in-person scales assuming the absence of distinctive factors of eHealth TA (Clarke et al., 2016; D'Alfonso et al., 2020; Ormrod et al., 2010).

To address these gaps, we developed the eHealth Therapeutic Alliance Inventory (ETAI) (Ashur et al., 2023), which incorporates subscales specifically designed to capture the unique facets of TA in the digital settings, alongside conventional TA typically applied in in-person therapy. This approach facilitates a quantitative examination of whether eHealth TA facets exist independently and how they relate to clinical outcomes, in comparison to conventional TA facets.

The ETAI includes three subscales that assess digital health-specific aspects of TA. The first, Sense of Relatedness, measures the extent to which the program enables the users to personify the digital program and form a connection with its therapeutic representative (e.g., a therapeutic bot), the platform as a whole, or the community within the program. The second, Application-Induced Accountability, reflects the degree to which the program fosters a sense of responsibility in users, motivating them to engage consistently with the therapeutic process. The third, Perceived Emotional Investment, captures the emotional significance users attach to their therapeutic goals and the emotional impact of achieving or failing to achieve those goals (Ashur et al., 2024). In addition to these digital health-specific subscales, the ETAI includes a Conventional TA subscale that offers a digitized version of Bordin's classic model of TA, which comprises three core components: Goal, Task, and Bond (Bordin, 1979).

Our previous studies have provided support for the existence of the unique eHealth TA facets as independent constructs, suggesting that these facets may have stronger predictive validity in digital health settings compared to traditional TA measures (Ashur et al., 2023, Ashur et al., 2024). However, these findings remain preliminary, highlighting the need for further investigation, particularly using diverse research methodologies beyond the quantitative approach utilized in previous studies.

### 1.2 Current study

The current study utilized qualitative data collected through interviews and participant's responses to open-ended questions following their use of a digital parent training program, to further explore and validate our earlier preliminary findings. Our analysis focused on participants' experiences with a digital health program and their perceptions of its effectiveness. The primary objective was to assess whether participants, in reflecting on their experiences, would naturally emphasize aspects of the therapeutic alliance that are distinctive to digital health, as identified in our prior research. We hypothesized that this qualitative approach would complement and reinforce our earlier quantitative findings, which indicated the presence of therapeutic alliance facets that are unique to digital settings.

## 2 Materials and methods

This study extends the work of Ashur et al., 2023, Ashur et al., 2024, which investigated the therapeutic alliance (TA) facets unique to digital health settings and their relationship to clinical outcomes. The study protocol was approved by the institutional review board at the University of Haifa (approval number: 058/22).

### 2.1 Participants and recruitment procedure

Parents were invited to participate in the study via a Facebook campaign conducted between May and July 2022. Those who submitted their contact information were asked to complete a brief eligibility screening questionnaire, which included inclusion and exclusion criteria, as well as questions about their child's behavior. Eligibility criteria for participation included: (1) being a parent of a child aged 3 to 7 years old who exhibited (2) elevated levels of problem behaviors as measured by the Eyberg Child Behavior Inventory (ECBI) subscales (ECBI-problem ≥15 or ECBI-intensity ≥132), and (3) possessing a smartphone with cellular and internet connectivity. Exclusion criteria included: (1) the child was currently receiving treatment for behavioral or emotional issues, or the parent was involved in another parent training program; and (2) the child had a diagnosis of intellectual disability or developmental delay. Eligible parents were contacted by phone to confirm their eligibility and provided additional details about the program and study. Interested parents were then asked to electronically sign a consent form and complete a baseline assessment. Parents whose children did not meet the eligibility criteria were directed to available public mental health services. Eligible parents were randomly assigned to either an enhanced-quality or a standard-quality program. Login information was sent to participants via email and text message. Although the two DPT programs differed in their quality (Baumel et al., 2023), the primary focus of this study was to explore participants' natural descriptions of the therapeutic alliance with the digital health program, irrespective of the program's specific characteristics. Therefore, we did not focus on comparing the differences between the two intervention groups.

### 2.2 Overview of interventions

The DPT programs utilized in this study are grounded in the principle that parents' actions and responses can significantly shape their child's behavior (Domhardt et al., 2021; Schmidt and Schimmelmann, 2015). Developed by Prof. Baumel, the DPTs integrate common strategies of parent training programs (Baumel et al., 2023), and includes seven modules, each focusing on a specific theme: (1) introduction to parent training; (2) positive interactions and quality time; (3) parental emotion regulation; (4) effective routines and clear ground rules; (5) recognizing positive behaviors and ignoring minor negative behaviors; (6) overcoming disobedience; and (7) mindful parenting and communication between partners (Baumel et al., 2023; Saar et al., 2024).

### 2.3 Measures

#### 2.3.1 Semi-structured interview

A semi-structured interview was conducted with 16 parents who participated in the study and used the program, focusing on their experiences and perceptions of the program's effectiveness. The interview, led by a psychologist via Zoom, aimed to capture the parents' unique experiences, particularly in balancing their daily family and work obligations. A standardized interview guide was used to ensure consistency across interviews. Parents were prompted to elaborate on their responses, share examples, or relate to program features relevant to their answers. The questions included: (1) Have you found yourself thinking about the skills or tools presented in the program while spending time with your child, such as during the afternoon or evening? Please share more about your experience.; (2) When you are with your child, for example, in the afternoon, how clear is it to you how to address or react to your child's behavior?; (3) How clear is the connection between the goals you had when joining the program and the skills and tools you acquired through the program?; (4) Did you feel that the program motivated you to change your parental behavior? If so, in what ways?; (5) Have you noticed a change in your availability or presence with your child since participating in the program? Please elaborate. From the perspective of the current study the interviews were conducted in a double-blind manner. Both the interviewer and the interviewee were unaware of the specific focus on the therapeutic alliance or the unique themes expected to emerge in digital health contexts. This approach helps reduce interviewer bias while ensuring that participants share their authentic experiences.

#### 2.3.2 Open-ended questions

As part of the post-intervention assessment, 72 parents responded to a series of open-ended questions administered via Qualtrics. Participants were encouraged to provide written responses without length restrictions, allowing for in-depth reflections on their experiences. The open-ended questions were: What advantages, if any, did you find in using the program to create a change in the way you react to your child?; Did the program assist you in creating a positive change with your child?; What aspects of the program did you appreciate the most?

### 2.4 Data analysis

In the analysis of the semi-structured interviews, we performed a thematic analysis of participant responses following the six-step framework outlined by Braun and Clarke (2006). Interviews were transcribed (by CRS), and the lead author initiated coding to identify preliminary themes, which were refined through iterative collaboration (with AB), resulting in a comprehensive theme map. We conducted a deductive thematic analysis following Braun & Clarke (2006, 2023), a common approach used to examine how theoretically derived constructs appear in qualitative data. Our analysis was informed by the themes of TA derived from our previous work on the eHealth Therapeutic Alliance Inventory (ETAI), incorporating both conventional and new aspects of digital TA. This process allowed us to determine which therapeutic alliance themes were reflected in participants' descriptions of their experiences with the program. In cases where support for a theme in participants' answers was partial, we noted that the findings were marginal and described the reasoning for this note. In the second phase of analysis, we examined responses to the open-ended questions from the post-intervention assessment. Out of the 72 parents who responded to the open-ended questions, 64 provided usable data, while 8 responses were excluded due to incomplete answers or minimal elaboration (e.g., stating that the program was helpful without further details). We analyzed these responses in relation to the themes identified in the first phase. This process allowed us to estimate the relative importance of each theme across a larger sample of parents who had engaged with the program. Although open-ended responses tend to be less detailed than interview data, we assumed they would provide valuable insights into the most salient aspects of the therapeutic alliance for participants. We reviewed each response, categorized content according to the four main themes, and calculated the percentage of parents who referenced each theme out of the total number of parents who responded to the open-ended questions.

## 3 Results

Demographic details for all participant groups are provided in Table 1. No significant differences were found between the interview participants and those who responded to the open-ended questions in terms of group characteristics.

**Table 1:** Participant demographic characteristics.

		Interview participants	Open-ended question participants
Continuous		M (SD)	M (SD)
Parent age (years)		37.13 (4.81)	36.31 (3.37)
Child age		4.96 (1.35)	4.94 (1.33)
Number of children in the family		2.44 (0.62)	2.64 (0.89)

Categorical		N (%)	N (%)
Leading parent gender	Female	15 (93.75 %)	62 (96.87 %)
	Male	1 (6.25 %)	2 (3.12 %)
Child gender	Female	5 (31.25 %)	27 (42.18 %)
	Male	11 (68.75 %)	37 (57.81 %)
Participating	Both parents	11 (68.75 %)	39 (60.93 %)
	One parent	5 (31.25 %)	25 (39.06 %)
Education a	High school	0	8 (12.50 %)
	Above	16 (100 %)	56 (87.50 %)
Household income b	< 15 K	1 (6.25 %)	9 (14.06 %)
	15–18 K	4 (25 %)	21 (32.81 %)
	> 18 K	11 (68.75 %)	34 (53.12 %)
Religiosity	Secular	10 (62.5 %)	36 (56.25 %)
	Traditional	4 (25 %)	18 (28.12 %)
	Religious	2 (12.5 %)	10 (15.62 %)
Hours of work/study per week a	> 10 h	1 (6.25 %)	9 (14.06 %)
	10–29 h	2 (12.5 %)	12 (18.75 %)
	> 30	13 (81.25 %)	43 (67.18 %)

a Refers to the parent leading the intervention;

b In Israeli shekels (ILS).

### 3.1 Thematic analysis of interview responses

**Fig. 1:**
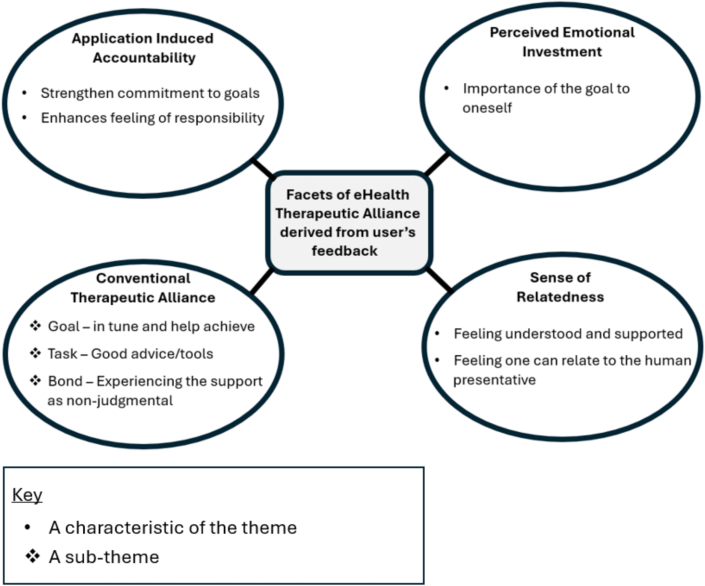
Map of themes and sub-themes.

Through deductive thematic analysis, the four main themes comprising ETAI were identified (see Fig. 1 for a visual representation of these themes, with sub-themes included where relevant). In this section, we provide a brief overview of each theme, supported by representative quotes from parents who participated in the interviews.

#### 3.1.1 First theme: conventional therapeutic alliance

This theme reflects the traditional understanding of the therapeutic alliance in in-person psychotherapy, with adaptations and translations to the digital context. It encompasses three sub-themes.

##### 3.1.1.1 Sub-theme 1 – goal

This sub-theme reflects the degree to which users align with the program's objectives and perceive it as effectively designed to help them achieve their desired outcomes. Users emphasized the importance of the program's relevance to their personal goals. For example, one parent shared, “*For me, it (the program) guided us smoothly to the place we, as parents, wanted to reach.*” Another parent noted, “*It helped us structure our parenting vision and provided clarity on its direction.*”

##### 3.1.1.2 Sub-theme 2 – task

This sub-theme represents the degree to which users perceive the tasks and activities within the program as relevant, effective, and aligned with therapeutic goals. The alignment of the program's tasks with individual objectives emerged as a central theme throughout the interviews, with participants emphasizing how the program's tasks effectively guided them toward progress and were tailored to their needs. One parent shared, “*I received coping strategies I hadn't used before… I found that using them helped reset everything immediately*”. Another parent noted, “*I appreciated the program's guidance to pause before reacting… taking a moment, then returning to the situation... it worked like magic*”.

##### 3.1.1.3 Sub-theme 3 – bond

This sub-theme reflects the quality of the emotional connection and trust that users feel toward the program. It highlights the extent to which participants experience support, understanding, and respect throughout the process, creating a sense of safety and collaboration. However, content related to emotional connection was limited, mainly focusing on non-judgmental and constructive feedback. One parent shared, “*I didn't feel judged by the program; instead, it communicated a reassuring message: ‘It's okay, making mistakes is normal, it's part of the process’*”. This was considered marginal, as users did not express a strong sense of connection to the program or discuss the development of trust in it.

#### 3.1.2 Second theme: application induced accountability

This theme reflects the extent to which the program instills in users a sense of accountability toward engaging in the therapeutic process. It highlights how the program not only strengthens users' sense of responsibility toward their progress but also reinforces their actions toward achieving therapeutic goals. For instance, one mother shared, “*The program reminded me that it's all up to me; every time, it would return responsibility and control back to me*”. Another parent stated, “*I have to say, the program motivated me. It made me think about things, they stayed in my awareness*”.

#### 3.1.3 Third theme: perceived emotional investment

This theme reflects the degree to which users invest emotionally in the therapeutic process, particularly driven by a fear of disappointing themselves or the program. Although support for this theme was considered marginal. Some participants did express the emotional significance they attached to their progress. One participant shared, “*I took it on as a real project; it was important to me, so I decided to give it a genuine chance and invest in it fully*”. Another participant noted, “*I'm really trying to do my best. It's important to me, so I'm always seeking out tools and knowledge*”. This was considered marginal, as users did not express a fear of disappointing themselves or the program but only expressed emotional investment in the therapeutic process.

#### 3.1.4 Forth theme: sense of relatedness

This theme reflects how easily users feel they relate to a human-like or relatable figure embedded within the digital program. It represents the degree to which users feel they are communicating with human representatives who offer support, even though they know the program is automated and self-guided. One participant shared, “*It kind of gave the feeling that… I can really relate to the team that created this program*”. Another participant noted, “*I also enjoyed the videos and really connected with the facilitator who presented them; it made the experience feel more personal*”.

### 3.2 Analysis of responses to open-ended questions

We analyzed 64 responses from users, with the results summarized in Table 2. Nearly 80 % of parents described aspects of their experience related to the task sub-theme, which is a part of the conventional therapeutic alliance theme. Over half (57.81 %) of the parents highlighted issues related to a sense of relatedness toward the program. Approximately one-third (32.81 %) mentioned aspects of accountability induced by the application; 10.93 % of parents referenced the goal sub-theme; and 6 % explicitly addressed perceived emotional investment. Notably, no parents explicitly mentioned a bond with the program.

**Table 2:** Results from open-ended questions: number of parents relating to each main theme or sub-theme (% out of total number of parents).

	Conventional TA Themes			Digital Health TA Themes		
	Goal	Task	Bond	AIA	PEI	SIR
All relevant users (*n* *=* *64)*	7 (10.93 %)	51 (79.68 %)	0	21 (32.81 %)	4 (6.25 %)	37 (57.81 %)

*Note.* Goal, Task and Bond are sub-themes of the Conventional Therapeutic Alliance theme; AIA – Application Induced Accountability; PEI – Perceived Emotional Investment; SIR – Sense of Relatedness.

## 4 Discussion

The primary aim of this study was to validate the preliminary quantitative findings from prior research regarding the unique facets of the TA in digital health settings through qualitative inquiry. Through thematic analysis of semi-structured interviews and analysis of open-ended survey responses, we gathered qualitative evidence that aligns with the eHealth TA themes identified in our previous studies (Ashur et al., 2023, Ashur et al., 2024). Specifically, the key eHealth TA themes – Application-Induced Accountability, Perceived Emotional Investment, and Sense of Relatedness – were reflected in participants' descriptions, highlighting the significance of these factors in digital health interventions. Additionally, conventional TA themes (such as Goal, Task, and Bond) also appeared, indicating that traditional aspects of TA remain relevant within digital settings.

However, the theme of Perceived Emotional Investment received only marginal support in the qualitative analysis. While some participants expressed emotional investment in the program, no one reported experiencing emotional disappointment or a sense of failure when not achieving their therapeutic goals – an essential component of this theme. Several factors may explain this discrepancy. First, digital health programs may not evoke the same emotional engagement as in-person therapy (Lipschitz et al., 2023; Baumel and Yom-Tov, 2018; Baumel, 2022; Michie et al., 2017; Hardiker and Grant, 2011). The digital format might result in participants engaging with the program in a less committed manner, leading to lower emotional investment. Additionally, the study's methodology might have played a role. While the double-blind design reduced bias, it may have limited the depth of inquiry into participants' emotional engagement. The interviewer did not specifically probe participants about their emotional investment or the impact of failure, which could explain the lack of support for this theme.

A similar pattern of marginal support was observed with the conventional TA theme, particularly within the Bond subscale. This subscale reflects the establishment of a trusting, empathetic relationship between participant and therapist or intervention. However, participants' responses tended to focus more on functional or task-oriented aspects rather than emotional connections. They viewed the program primarily as a tool for self-improvement, rather than something fostering deep emotional rapport. The absence of human interaction may have hindered the development of a deeper emotional bond with the program, weakening the sense of “bonding.”

This difficulty in validating the Bond theme in digital health settings reflects ongoing challenges in translating aspects of in-person therapeutic alliances to digital formats. Prior research on eHealth TA measurements suggests that simply replacing “the therapist” with “the app” or “the program” can lead to awkward phrasing (e.g., “The program and I respect each other”), which might cause clients to anthropomorphize the program or assume emotional connections that aren't truly present in digital health contexts (Berry et al., 2018; Goldberg et al., 2021). This aligns with the work of Weinberg et al. (2022), who advocate for exploring the unique form of intimacy that develops in digital settings – referred to as “e-ntimacy”, rather than expecting traditional emotional intimacy to naturally emerge in digital health settings.

From a methodological standpoint, we believe that our approach of combining quantitative analysis with qualitative validation offers a robust framework for this area of research. This methodology contrasts with earlier studies on eHealth TA, which either qualitatively explored interventions and theoretical models of eHealth TA (Holter et al., 2016, Holter et al., 2019; Brandt et al., 2013) or relied primarily on quantitative methods (Bickmore and Picard, 2005; Meyer et al., 2015; Ormrod et al., 2010).

### 4.1 Limitations

This study has several limitations. Methodologically, although the double-blind approach reinforced validity by minimizing interviewer and interviewee bias, it has limited the depth of inquiry into certain issues, as previously noted. Additionally, while the double-blind approach is commonly used in randomized controlled trials (RCTs) (Crisp, 2015; Day and Altman, 2000; Miller and Stewart, 2011; Schulz and Grimes, 2002), we found no evidence of its use in qualitative psychology studies where both the interviewer and interviewee are unaware of the true aim of the semi-structured interview. This raises questions about its effectiveness in qualitative research settings.

Another limitation is the retrospective nature of the participants' responses, which introduces the potential for recall bias. Participants were asked to reflect on their experiences after completing the program, which could have influenced how they described their emotional engagement and perceived connections.

Moreover, while thematic analysis was employed, it remains a subjective process. Different researchers might interpret the same data in various ways. Despite efforts to ensure consistency, the inherent subjectivity of qualitative research means the findings are open to interpretation and may not be fully replicable in future studies. Future research could enhance reliability by using multiple coders or alternative methods such as member checking (McKim, 2023).

Also, a key limitation of the current study is that the sample was restricted to parents of young children in Israel, predominantly mothers whose educational levels are typical for the Israeli population but relatively high in comparison to global averages, which may constrain the generalizability of the findings. Future studies should examine whether the ETAI facets generalize to other populations, including different clinical groups, cultural contexts, and both guided and unguided interventions.

Lastly, because the qualitative analysis was primarily deductive, it is possible that certain themes that might have emerged in a more exploratory analysis did not surface, given our focus on the predefined theoretical framework. Future studies may choose to incorporate complementary inductive analyses to expand the range of potential emergent themes. This strategy may also help distinguish between findings that reflect genuine characteristics of digital therapeutic settings and those that may be influenced by methodological constraints.

### 4.2 Conclusions

Despite limitations, the study contributes to a deeper understanding of digital therapeutic dynamics. It calls for further exploration of digital health-specific therapeutic processes, highlighting the need for a more nuanced understanding of how therapeutic alliance unfolds in digital settings. From a methodological perspective, conducting quantitative analysis followed by qualitative validation offers a unique and robust framework for this area of research.

## Declaration of competing interest

The authors declare the following financial interests/personal relationships which may be considered as potential competing interests: Amit Baumel reports being a board member in the International Society for Research on Internet Interventions. He received grant funding as a principle investigator from 10.13039/501100003977Israel Science Foundation and 10.13039/501100003976The Israel National Institute for Health Policy Research. The funders had no role in the design, data collection, analysis, or preparation of the manuscript. Amit Baumel has provided consultancy to “ifeel” (which has products unrelated to the intervention or target population examined in this study). All other authors declare that they have no known competing financial interests or personal relationships that could have appeared to influence the work reported in this paper.

## Figures and Tables

**Figure:**
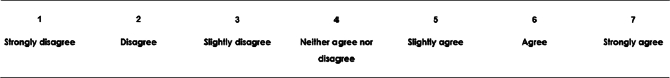


**Figure:**
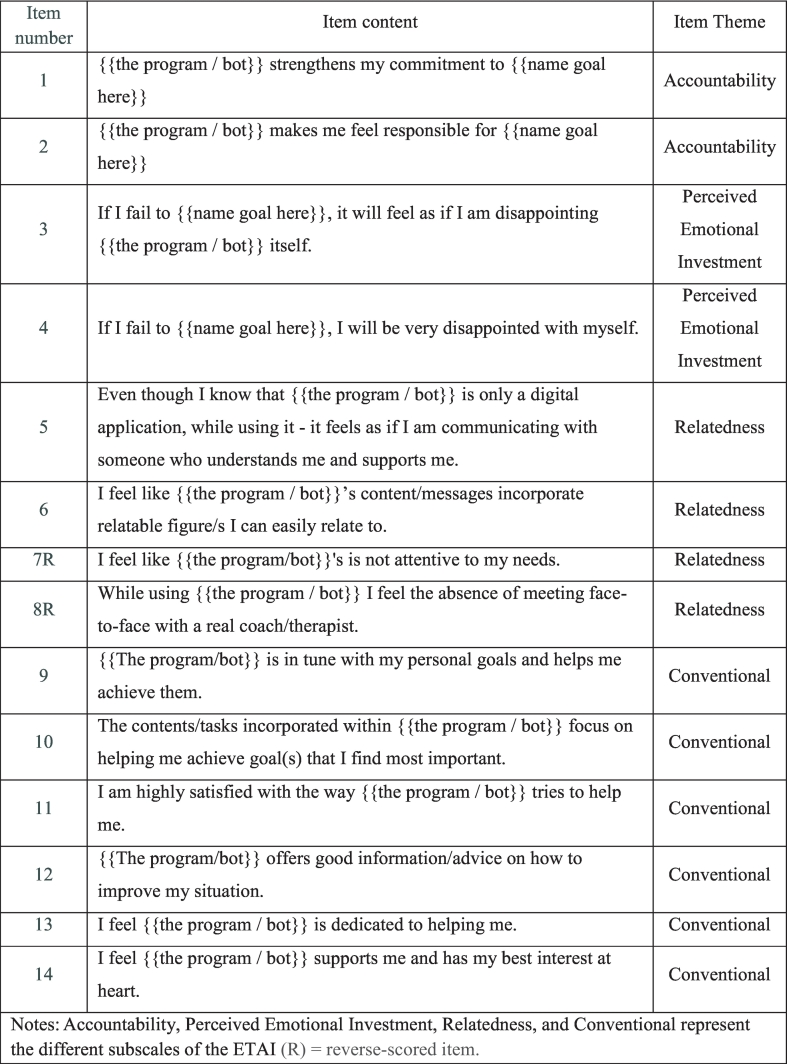


## Appendix A eHealth Therapeutic Alliance Inventory

Instructions:

Below is a list of statements that describe some of the different ways people might have thought or felt about the eHealth program they were using.

Please carefully consider your experience with the eHealth program you use (or have used in the past), and then for each statement circle the number indicating how strongly you agree or disagree, on a seven-point scale:

If the statement strongly describes the way you feel (or think) circle the number 7; if the statement strongly contradicts the way you feel (or think) circle the number 1.

Use the numbers in between to describe the variations between these extremes.

Please rate **every** statement.

Reminder: This questionnaire is confidential. Only the research team will see your answers.

Thank you for your cooperation.

Notes: Accountability, Perceived Emotional Investment, Relatedness, and Conventional represent the different subscales of the ETAI (R) = reverse-scored item.
